# Patient-reported outcomes in cancer survivorship: insights from two decades of population-based PROFILES registry research

**DOI:** 10.1007/s11764-024-01690-4

**Published:** 2024-10-08

**Authors:** Floortje Mols, Dounya Schoormans, Simone Oerlemans, Nicole Horevoorts, Nicole Ezendam, Natasja Raijmakers, Lonneke van de Poll-Franse

**Affiliations:** 1 CoRPS - Center of Research on Psychological Disorders and Somatic Diseases, Department of Medical and Clinical Psychology, Tilburg University, PO Box 90153, 5000 LE Tilburg, The Netherlands; 2 Department of Research & Development, Netherlands Comprehensive Cancer Organisation (IKNL), Utrecht, The Netherlands; 3 Division of Psychosocial Research and Epidemiology, Netherlands Cancer Institute, Amsterdam, The Netherlands

**Keywords:** Cancer, Survivorship, Registry, Patient-reported outcomes, PROFILES

## Abstract

**Purpose:**

When the field of cancer survivorship research was in its infancy, the PROFILES registry was set up in 2004 to monitor patient-reported outcomes (PROs) in survivors and a normative population. This scoping review aims to summarize lessons learned from developing a population-based PRO registry, focusing on study methodologies, data collection shifts, data utilization, multidisciplinary collaboration, societal impact, and data sharing.

**Methods:**

A systematic computerized literature search through PubMed was performed to collect all publications using data from the PROFILES registry between January 1, 2004, and December 31, 2023.

**Results:**

The PROFILES registry’s research today encompassed 249 papers from 35 studies. Key insights include the importance of multi-hospital collaboration, which enhances participant inclusion and result generalizability. Optimizing response rates and patient inclusion is achieved through proactive data collection methods such as inclusion by health care professionals, and using both web-based and paper questionnaires. Longitudinal studies, despite their intensive data collection efforts, provide critical insights into the consequences of cancer and its treatment on patient-reported outcomes (PROs) from diagnosis through survivorship. Combining PRO data with comprehensive clinical registry data ensures reliable datasets, crucial for drawing meaningful conclusions. The shift towards multidisciplinary collaboration, open-access publishing, and data sharing all contribute to accessible and impactful research.

**Conclusions:**

This review highlights key insights from the PROFILES registry, emphasizing multi-hospital collaboration, proactive data collection, and the integration of PROs with clinical data.

**Implications for Cancer Survivors:**

These lessons can guide future research on cancer survivorship, improving methodologies to enhance survivorship care and quality of life through multidisciplinary collaboration and data sharing.

## Introduction

PROFILES (Patient-Reported Outcomes Following Initial Treatment and Long-Term Evaluation of Survivorship) is a population-based registry that was designed to collect data from cancer survivors and controls [1, 2]. It aims to describe and understand the possible impact of cancer and its treatment, beyond “normal” aging and the presence of co-morbidities. More specifically, it aims to study the physical and psychosocial impact of cancer and its treatment from diagnosis into survivorship, in different groups of survivors, including adolescents and young adults (AYA’s) or older patients, those with comorbidities, rare cancers, and those in the palliative stage. Within PROFILES, we use the American Cancer Society’s definition of cancer survivorship, which defines cancer survivors as those living with, through, and beyond cancer.

At the beginning of the twenty-first century, the Eindhoven Cancer Registry (ECR), which is now part of the Netherlands Cancer Registry (NCR) [3], celebrated its 50th anniversary. As one of the oldest cancer registries in the world, we were proud of its achievements and the significant impact it has had on patient care [3]. However, we recognized a critical gap: despite having very detailed clinical information on *all* newly diagnosed patients with cancer in the Netherlands, we lacked data on their well-being. At that time, the field of cancer survivorship was still emerging globally [4]. Concurrently, there was a growing international interest in understanding the long-term effects of cancer treatment at a population level, driven by organizations like the Dutch Cancer Society and the European Organisation for Research and Treatment of Cancer (EORTC). This insight was essential for preparing future cancer care strategies worldwide.

In 2004, we started with four population-based cross-sectional studies on the health-related quality of life (HRQoL) in long-term survivors of prostate cancer, endometrial cancer, and Hodgkin’s and non-Hodgkin’s lymphoma [5–8]. In these studies, we selected large cohorts of long-term cancer survivors from the ECR and sent them a simple paper-and-pencil questionnaire. From these pioneering studies, we learned that there was a great need and enthusiasm for this research among both survivors and health care professionals. However, we also learned that sending paper-and-pencil questionnaires to large cohorts of survivors, in multiple hospitals at the same time, by hand, was inefficient. In addition, as survivors raised their voices and asked for online questionnaires, we felt a strong need to be able to offer them an online version of our questionnaires alongside the paper-and-pencil version. Longitudinal studies with dynamic patient inclusion (i.e., approach newly diagnosed patients with cancer on a daily basis in order to include them before treatment) were also high on our wish list, but the administrative burden would be even higher. All these ambitions led to the official establishment of the PROFILES registry in 2009 [1].

The field of cancer survivorship has grown over the years, not only in terms of the prevalence of cancer survivors, the number of studies performed, and the number of researchers involved, but also in terms of the large range of topics studied [9], with multidisciplinary research becoming the norm. The PROFILES registry has experienced similar growth. There are currently over 770,000 people living with and after cancer in the Netherlands. They have been diagnosed with cancer sometime in the last 20 years. Over the years, more than 30,000 cancer survivors have participated in our studies. In order to be able to keep up with that progression, the registry was expanded with novel features from 2016 onwards. Since then, information from questionnaires and the NCR was enriched with novel, ambulatory, and objective measures (i.e., activity trackers, blood draws, hair samples, food diaries, online cognitive tests, and weighing scales) to create a multidomain data source for mechanistic cancer survivorship research [10]. Numerous studies are now making use of these extended measurement features within the PROFILES registry [11].

In the near future, we will publish our unique multi-data research, but before we do that, it is time to look back and reflect on the period when we merely focused on patient-reported outcome (PRO) measures assessed by questionnaires, enriched with clinical data from the NCR. The value of these studies is probably greater than the sum of their parts, and valuable lessons can be learned from those studies. Therefore, the aim of this scoping review is to summarize our lessons learned from developing a population-based PRO registry by investigating and reflecting on what the last 20 years taught us about: (1) the methodology of our studies with respect to the expansion of patient inclusion in a few regional hospitals to hospitals throughout the Netherlands, the shift from cross-sectional to longitudinal studies to randomized controlled trials (RCTs), and different approaches of including patients; (2) the shift from paper-and-pencil questionnaires to online questionnaires, the concepts being measured, and the questionnaires being used; (3) the value of using normative data, combining datasets, and including clinical cancer registry data; (4) the increase in the number of multidisciplinary publications over the years; (5) the societal impact of the studies; and (6) the value of data sharing. In short, this paper will focus on our lessons learned.

## Methods

### Design

The design of a scoping review was used since scoping reviews are exploratory and typically address a broad question. With this scoping review, we explored all publications using data from the PROFILES registry to examine possible lessons learned. Since PROFILES registry researchers were involved in all of these publications, we essentially reviewed our own work to draw conclusions and identify lessons learned.

### Search strategy

A computerized search of the literature through the search engine PubMed was performed on December 31, 2023. The search terms included concepts of “PROFILES registry” and “cancer,” in combination with “name of PROFILES researcher.” We searched for publications published between January 1, 2004, and December 31, 2023. All search results related to the various PROFILES researchers were imported into Covidence, which was used to remove duplicates. Articles were screened by title and abstract according to the pre-specified inclusion and exclusion criteria (see below). Full-text papers were then retrieved and checked for eligibility. Publications that did not meet the eligibility criteria were removed. The reference lists of all publications that met the eligibility criteria were also checked to retrieve other relevant publications not identified by means of the computerized search.

### Inclusion and exclusion criteria

Publications that met the following criteria were included: (1) if the objective was to describe the results of studies performed with the PROFILES registry, (2) if the publication was an original article (e.g., no poster abstracts, editorials, reviews, protocol papers, book chapters, and letters to the editor), (3) if the manuscript was published in peer review journals, and (4) if it was written in English. Publications of external researchers that made use of the PROFILES registry for their own data collection purposes are beyond the scope of this review as these publications can be difficult to retrieve and are often different in setup and purpose compared to our PROFILES publications. In addition, some publications only described the normative (non-cancer) data of the PROFILES registry to compare it with other (not PROFILES) patient data. In that case, the manuscripts were also not included in this review. Original PROFILES registry manuscripts on the normative population or comparisons of the normative population with PROFILES data on survivors were included. Finally, some studies examined the perspective of health care providers instead of the perspective of survivors. These publications were excluded from this review.

## Results

### Characteristics of the studies

Between January 2004 and December 2023, 249 papers were published using PROFILES registry data that met our selection criteria. These papers belong to a total of 35 studies. The mean number of publications per study was 5 (range 1–37). Most publications included survivors diagnosed with colorectal cancer (*n* = 84), followed by endometrial cancer (*n* = 44), prostate cancer (*n* = 35), thyroid cancer (*n* = 26), multiple myeloma (*n* = 24), ovarian cancer (*n* = 22), and non-Hodgkin’s lymphoma (*n* = 22). The response rates of the studies were not always calculated similarly. However, overall rates varied between 20% [12] and 86% [13]. Studies with relatively low response rates were studies among relatives of survivors [14], RCTs [15, 16], a study among AYAs [17], and a study in which survivors were invited to participate by the hospital administration instead of their own health care provider [18]. Studies with high response rates were often conducted in the earlier years. The number of survivors included in the studies varied between 18 [19] (qualitative study) and 4094 (quantitative study) [20]. The studies included in this review collected data between 2003 [21] and 2021 [22]. The survivors included in the publications were diagnosed between 1989 [7] and 2019 [23].

### Methodology of the studies

Over the years, the number of hospitals that participated in PROFILES studies expanded from hospitals in the south of the Netherlands, where the PROFILES registry started, throughout the Netherlands as a whole including all general and academic hospitals.

Although the initial PROFILES studies were cross-sectional [24], over the years, this shifted towards longitudinal studies [25]. In total, 22 studies were cross-sectional, 10 were longitudinal, and 3 were RCTs. In total, 179 cross-sectional papers were written including cross-sectional analyses on longitudinal datasets, and 67 longitudinal papers were published. Often, findings from our cross-sectional studies, for instance on colorectal cancer [26], thyroid cancer [13], breast cancer [21], prostate cancer [27], Hodgkin’s lymphoma [7], non-Hodgkin’s lymphoma [28], chronic lymphocytic leukemia [29], and multiple myeloma [30], lead to setting up longitudinal studies addressing additional questions that arose from our previous work [11, 25, 31, 32]. Moreover, some observational studies (i.e., on gynecological tumors, lymphoma, and chemotherapy-induced peripheral neuropathy (CIPN)) led to the design of RCTs in order to have a direct impact on patient care [33–35]. For instance, we published numerous studies on the influence that CIPN can have on patients’ lives [36–39]. These results led to an RCT that will provide valuable information on the effectiveness of an online self-help intervention based on acceptance and commitment therapy versus a waitlist control group for survivors with chronic painful CIPN [33].

The majority of publications (*n* = 213, 86%) were based on studies that enrolled survivors using the NCR as a sampling frame (i.e., a selection of survivors from the NCR received a letter from their (ex-) attending specialist asking them to participate) [5]. These studies mainly included long-term survivors years after diagnosis. In more recent years, inclusion shifted towards health care professionals asking survivors directly during face-to-face consultations, allowing survivors to be included directly after diagnosis [40]. In the discussion, we will describe the lessons learned regarding the methodology of the studies.

### Measures

The oldest studies used paper-and-pencil questionnaires only, but from 2009 onwards, most studies included both online and paper-and-pencil questionnaires. From 2017 onwards, some PROFILES studies solely included online questionnaires. These were studies on the value of internet use [12] and online applications [41, 42], studies that recruited survivors online [43, 44], and a study performed in the first lock-down of the COVID-19 pandemic [20]. Our normative population questionnaires have been fully online since 2009 [45–47].

The oldest PROFILES studies focussed mostly on HRQoL, disease-specific symptoms, and health care utilization [6–8, 27, 48, 49]. Over the years, the scope broadened to a wide range of PROs like psychosocial outcomes (e.g., anxiety [38], depression [50], optimism [51], adjustment disorders [16], personality [52], illness perception [53], coping [54], mindfulness [39], and fear of cancer recurrence [55]), clinical outcomes (e.g., mortality [56, 57], prognosis [58], comorbidity [59], recurrent [60], and second cancers [61]), lifestyle (e.g., physical activity [62], sleep [63], BMI [64], food intake [65], and alcohol consumption [66]), side effects of treatment (e.g., fatigue [67], CIPN [36], alopecia [68], ostomies [69], cognitive functioning [70]), health care (e.g. unmet needs [71], adherence [72], health care use [73], supportive care [74], follow-up care [75], diagnostic intervals [76], dietary support [77], self-management [72]), information provision (e.g., satisfaction with information [78], internet use [12], survivorship care plans [79], health literacy [80]), and the financial impact of cancer (i.e., SES [81], financial toxicity [82], employment [83], and insurance [84]). Also, we expanded from studies on cancer survivors to studies on relatives of survivors with cancer [85]. Finally, a trend towards assessment of more PROs per study resulted in longer questionnaires compared to the early days.

The validated EORTC QLQ-C30 [86] was most often used to assess HRQoL, a variety of disease-specific EORTC questionnaires were used to assess disease-specific QOL, the EORTC QLQ-CIPN20 [87] to assess CIPN, the EORTC QLQ-INFO26 for information provision [88], the HADS [89] for anxiety and depressive symptoms, the FAS [90] or MFI [91] for fatigue, the Pittsburgh Sleep Quality Index for sleep quality [92], the EPIC [93] or SQUASH [94] for physical activity, the DS14 [95] for personality, and the adapted Charlson Comorbidity Index [96] to assess comorbidity. In some areas, validated questionnaires did not exist, or did not exist in Dutch, so they were self-developed. The topics related to these questionnaires most often concerned employment, insurance, health care utilization, internet use, and lifestyle (smoking, alcohol use, BMI).

### Combining data

In 18% (*n* = 49) of the publications, data from multiple studies were combined. Examples include studies on health care utilization, information provision, financial impact, illness perception, and employment, and studies on the impact of depression on mortality. Furthermore, in 26% of the publications (*n* = 63), the studied patient population was compared with an age- and sex-matched normative population. In all PROFILES studies, PRO data from cancer survivors were combined with clinical data from the NCR.

### Publications

**Fig. 1:**
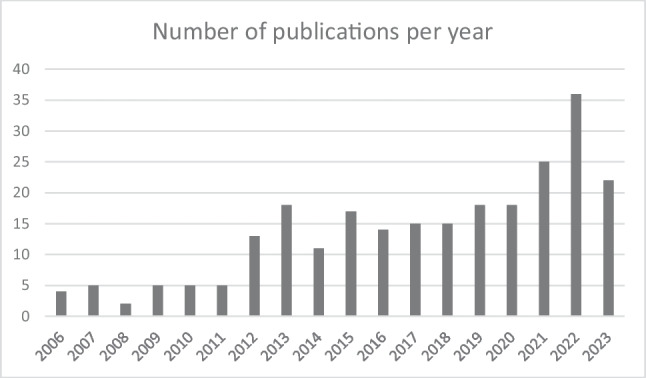
Number of publications per year

The number of published research articles grew over the years (Fig. 1). Most of the PROFILES publications (*n* = 175, 71%) were published open access. All publications except one [97] were truly multidisciplinary since they were a collaboration between researchers from at least two, but most often, three or more different backgrounds, like physicians, psychologists, and medical specialists.

### Societal impact

Studies using PROMS can have an impact on health care and the everyday lives of cancer survivors. Some publications had more impact than others, and describing the impact of all papers goes beyond the aim of this scoping review. However, we will give a few examples of papers that have had a clear impact. For instance, a study on the socio-economic impacts of cancer showed that a significant proportion of survivors experienced changes in their employment status and faced difficulties in obtaining health and life insurance and home loans [84]. This paper received press attention, parliamentary questions were raised, and now, years later, and thanks in part to intensive lobbying of a cancer patient organization, the “clean slate rule” was introduced making it possible for more survivors to take out a mortgage after being diagnosed with cancer. Second, a paper investigated the long-term side effects of (R)-CHOP (rituximab, cyclophosphamide, doxorubicin, vincristine, and prednisone) treatment every 2 weeks ((R)-CHOP14) versus every 3 weeks ((R)-CHOP21) in survivors with diffuse large B-cell lymphoma [98]. Findings indicated that survivors who underwent (R)-CHOP14 more often reported tingling in hands and feet, were more often fatigued, and had more often a slowed down feeling compared to survivors treated with (R)-CHOP21. With respect to overall survival, it was thought that CHOP every 14 days was superior to a 21-day schedule. Together with two RCTs showing no overall survival difference between (R)-CHOP14 and (R)-CHOP21, the findings of our study contributed to a change in guidelines from (R)-CHOP14 to (R)-CHOP21 as the preferred standard first-line treatment for survivors with DLBCL. Third, the eQuipe study is focused on evaluating the quality of end-of-life care among cancer survivors [99]. The results of this study have contributed to a greater awareness of the need for early integration of palliative care into oncology. National recommendations for this early integration are now in progress. Fourth, a population-based study among colorectal cancer survivors raised awareness about the chronic nature of chemotherapy-induced neuropathy and its huge negative impact on HRQoL [36]. It underscored the need for improved patient care strategies including better management of neuropathy symptoms, development of neuroprotective strategies, and provision of comprehensive support to improve the HRQoL of survivors. The study’s insights led to more studies in this area which eventually led to the halving of the dose of chemotherapy for these survivors. Finally, the Lymphoma Intervention (LIVE) trial had an impact on patient engagement in health care [42]. Since the return of individual PROs to lymphoma survivors seems to meet survivors’ wishes and had no negative impact on psychological distress, self-management, satisfaction with information provision, and health care utilization, the return of individual PROs can be safely implemented in daily clinical practice.

### Data sharing

Since 2009, the PROFILES data has been available upon request for non-commercial scientific research for researchers worldwide. The data requests mostly came from the Netherlands. Also, data was shared with researchers from the USA, Australia, New Zealand, Norway, Belgium, Singapore, and the UK. In numerous cases, data dissemination led to publications that otherwise would not have been written since these researchers usually look at the PROFILES data from a different perspective or background, such as different health care systems. Over the years, the number of data requests has been quite stable. Finally, all PROFILES data are shared with survivors through a Dutch website for cancer survivors so that they can compare their PRO scores to scores of patients with a comparable age and cancer site (https://www.kanker.nl/kankersoorten/baarmoederkanker/gevolgen/gevolgen-van-baarmoederkanker).

## Discussion

In total, 249 PROFILES papers were published based on 35 PROFILES studies. The studies showed a wide range of included patient groups including highly prevalent cancers like breast, prostate, and colorectal but also the more rare cancers like thyroid cancer, gynecological cancers, hematological malignancies, and sarcomas. Besides studies on specific cancer groups, some studies focussed on other groups like AYA’s, those with advanced cancer, and relatives of those with cancer. Below, we provide the insights that we gained from those studies assessing PROs in cancer survivorship research over the past two decades.

### The importance of collaborating with numerous hospitals

Over the years, the number of hospitals that participated in PROFILES studies expanded from the southern part of the Netherlands where the PROFILES registry started, towards the Netherlands as a whole. Being able to perform studies in multiple hospitals at the same time enhanced the inclusion of possible participants, especially in rare cancers [11]. Also, it is easier to extrapolate the results from those studies to clinical practice since it is more likely that the included survivors are a true representation of the cancer population at large. Finally, translating the results of a study into clinical practice is more likely if more hospitals, and thus more health care professionals, are actively involved in a study.

### How to achieve optimal response rates and patient inclusion

The response rates of the PROFILES studies varied between 20 and 86%. The studies in which the NCR was used to make a selection of survivors who consequently received an invitation letter for a study on behalf of their (ex-) attending specialist often resulted in the highest response rates. With this method of data collection, the responsibility and workload of data collection belong completely to the researcher. Also, these studies focused on survivors > 1 year after diagnosis instead of including those right after diagnosis during a turbulent time in their lives as data becomes available in the NCR after a year. Furthermore, these studies were performed between 10 and 20 years ago when the field of cancer survivorship was still in its infancy, and survivors were maybe more willing to participate. Indeed, we noticed a decline in response rates of our studies over the years. Besides the fact that our data collection shifted towards earlier in the cancer treatment trajectory up to right after diagnosis, this can also be explained by the fact that the field of cancer survivorship has grown tremendously in the past two decades. This led to many competing studies for survivors to choose from on the one hand, and health care professionals were reluctant to offer too many studies to survivors considering the burden for the survivor on the other hand.

Besides response rates, the number of survivors included in PROFILES studies varied strongly (i.e. between 18 and 4094 for qualitative and quantitative studies respectively). Studies with a high number of included survivors were often studies on highly prevalent cancers, studies in which many hospitals participated, studies with a very proactive way of including survivors (e.g., additional phone calls from the research team to non-respondents), studies that offered both online and paper-and-pencil questionnaires, and studies that kept the burden for health care professionals very low.

### The struggle and value of longitudinal studies

Methodologically, the focus in cancer survivorship research has shifted from cross-sectional studies among long-term survivors towards longitudinal studies that follow survivors from diagnosis into survivorship. The only way in which PROFILES survivors can be included right after diagnosis is if health care professionals approach survivors directly during a face-to-face consultation. However, the time investment from health care professionals, researchers, and research assistants is significantly higher in those studies compared to studies that used the NCR as a sampling frame after which researchers send out single questionnaires to that specific selection of survivors. Also, including survivors right after diagnosis led to a drop in response rates, probably because survivors are preoccupied with treatment and the possible side effects of treatment. Furthermore, it is likely that not all eligible survivors are asked to participate in our studies since including them takes time from health care professionals. This might lead to selection bias in which only certain survivors are asked to participate.

Despite the lower response rates and more intensive way of data collection, longitudinal studies and RCTs are very valuable. The shift from cross-sectional to longitudinal studies led to more insight into the course and persistence of PROs during the survivorship trajectory from diagnosis until years later. This knowledge can be used by clinicians to make informed shared decisions regarding treatment, significantly improve screening for physical and psychosocial problems, make personal follow-up plans, and finally, develop and provide tailor-made treatments and supportive care interventions to optimize physical and psychosocial outcomes among cancer survivors.

### The value of adding population-based cancer registry data to PRO data

In all PROFILES studies, PROs were combined with clinical data from the NCR. As these data are collected by trained registrars among *all* survivors diagnosed with cancer in the Netherlands, these data are complete and reliable [100]. Also, this clinical NCR data enables us to combine data sets from cancer survivors since the clinical data belonging to those datasets is very comparable. Being able to have complete, reliable, and comparable clinical data across all PROFILES studies is something that often distinguishes PROFILES studies from other survivorship studies and underlines the value of core outcome sets [101].

### The valuable combination of both online and paper questionnaires

A shift was made from studies solely using paper-and-pencil questionnaires to studies using a combination of paper-and-pencil and web-based questionnaires in order to boost response rates by trying to meet the wishes of the individual survivor. Adding web-based questionnaires next to paper-and-pencil questionnaires led to less missing data and less ambiguous data (e.g., participants crossing multiple, implausible answer categories on paper). A comparison within PROFILES between a group that received a web-based questionnaire with the option to ask for a paper-based questionnaire, and a group that received a web-based questionnaire together with a paper-based questionnaire, showed no difference in response rates between these invitation modes [102]. This implies that it is better for researchers to leave out a paper questionnaire at invitation since it will save costs and increase precision. Since part of our (mainly elderly) respondents still prefer paper questionnaires, leaving them out completely is not an option yet [102].

### How to choose the most suitable PROMs

The oldest PROFILES studies focussed mostly on measuring HRQoL, symptoms, and health care utilization, but the scope broadened towards a very wide range of PROs with a trend towards more outcomes per study. This led to more and also broader knowledge on the impact of cancer and its treatment on the lives of cancer survivors. Also, our focus broadened from studies on cancer survivors to studies on relatives of cancer survivors. These expansions were often based on the trends in cancer survivorship research and the needs and preferences of both survivors and health care professionals.

A wide range of measurements was used in PROFILES studies. Besides the importance of choosing questionnaires with a high validity and reliability among cancer survivors, that are available in the right language, it proved to be useful to choose commonly used questionnaires to enable comparison between our results and the available scientific literature. It thereby improved the comparability of research, thus increasing the relevance and impact of the research findings.

### The value of comparability between studies

In a fifth of publications, data from multiple studies among cancer survivors were combined. Combining studies enabled us to draw conclusions on cancer survivorship on a broader level [48–50, 52, 53, 56, 67, 70, 82]. Furthermore, combining datasets enhances generalizability, increases statistical power, and enables subgroup analyses like the study of similarities and differences across cancer types (e.g., rare vs. common), and also similarities and differences regarding other factors like sex and treatment received.

This comparison was possible since almost all PROFILES studies used the same method to select and include survivors, used the same core outcome set, and in general had the same assessment times. In sum, being able to combine data from multiple studies is a valuable addition that distinguishes a registry from individual studies. Its results can advance scientific knowledge, improve clinical practice, and provide a strong evidence base for clinical guidelines and policy decisions. Therefore, making sure that studies are comparable with other studies is an important lesson learned.

### The added value of normative data

In a quarter of publications, PRO data of cancer survivors was combined with PRO data of age- and sex-matched normative population in order to evaluate the true impact of cancer and its treatment beyond aging and comorbid conditions. This has proven to be very insightful for the interpretation of the results of survivors and for clinical practice. For example, a study reported a better HRQoL among younger versus older survivors with diffuse large B cell lymphoma [103]. However, a comparison of HRQoL between these survivors and an age- and sex-matched normative population revealed that the differences found between younger survivors and the normative population were much larger than the differences between older survivors and the normative population. This suggests that having diffuse large B cell lymphoma has a greater impact on younger than on older survivors and that the lower HRQoL in older survivors in comparison with younger survivors is caused mostly by age itself and not by the disease and its treatment.

### Multidisciplinary collaboration is key

Almost all PROFILES studies (99%) are the result of multidisciplinary collaboration. Therefore, the content of our publications is often very much in line with relevant issues in both the scientific community and clinical practice as scientists work closely together with medical specialists. Also, collaboration between disciplines helps in translating scientific results into clinical practice. Although collaboration between different disciplines can be challenging, it often leads to results that are more valuable than the sum of its parts. For instance, researchers, psychologists, and medical specialists worked closely together on papers on anxiety, depression, illness perception, and personality related to clinical outcomes among cancer survivors [38, 104–107].

### The importance of open-access publications

The number of PROFILES publications grew tremendously over the years, as was to be expected considering the fact that the total number of performed studies increased and the number of PROs studied within these studies increased, and the fact that data of these studies were often merged in order to study general concepts on a broader level. However, having a registry with a high scientific output does not necessarily mean that its knowledge is available to those interested. Therefore, PROFILES researchers in general support the Berlin Declaration on Open Access to Knowledge in the Sciences and Humanities [108]. Currently, about two-thirds of PROFILES publications are publicly available, and this trend is getting stronger as open-access publications increase the accessibility and visibility of research results, thereby enhancing the possible impact that publications can have on improving care for cancer survivors.

### The power of sharing

All data from the PROFILES registry is available for non-commercial scientific research, subject to privacy and confidentiality restrictions. The data is cleaned, coded, and provided with metadata to ensure it can be easily accessed and understood by researchers. Initial analyses are performed to verify data quality and validity. After this process, the data is freely accessible for research inquiries from non-commercial groups upon registration on our website (www.profilesregistry.nl).

Dissemination of data has always been important to us, but there is still room for improvement. By increasing the findability of our datasets, and by simplifying our procedure for data transfer, we hope that more researchers will use PROFILES data in the near future. This will not only improve care for cancer survivors but using available data can also decrease the burden for cancer survivors participating in otherwise additional research and is therefore an efficient way to spend research funds.

Besides sharing data with researchers, we also share our data with cancer survivors on a Dutch website (https://www.kanker.nl/kankersoorten/baarmoederkanker/gevolgen/gevolgen-van-baarmoederkanker). In doing so, survivors can compare their PRO scores with those with a comparable age and cancer site. Broadening our perspective from scientific research to patient empowerment was a relatively effortless but valuable step.

Furthermore, we also share the PROFILES registry application itself. By sharing the PROFILES registry, we enable other researchers to collect their data in an efficient manner. The publications that resulted from other research groups using the PROFILES registry application were beyond the scope of this review. Examples of those studies are the 3P initiative (PACAP, POCOP, PLCRC) [109], umbrella [110], and Medias trial [111].

### Looking forward

New longitudinal PROFILES studies are currently running and besides the usual PRO data, these studies include novel, ambulatory, and objective measures (e.g., activity trackers, blood draws, hair samples, online food diaries, online cognitive tests, weighing scales, online symptoms assessment) [2] in combination with cancer registry data and normative data from a reference population. With these studies, we hope to contribute to better predictions of who will experience changes in PROs after cancer and why. This knowledge is needed to prevent and treat the long-term and late effects of cancer and its treatment and to develop interventions that promote health.

## Conclusion

This scoping review highlights the critical insights gained from nearly two decades of research utilizing the PROFILES registry. The registry’s evolution underscores the importance of comprehensive methodologies, including the integration of multi-hospital collaboration, proactive data collection approaches, and the combination of PROs with clinical data. These lessons can shape future research by promoting best practices in longitudinal data collection and ensuring that patient voices are effectively captured throughout the cancer survivorship journey. This review also emphasizes the growing significance of multidisciplinary collaboration and open-access data sharing in creating more accessible, and impactful research. Future studies should continue to explore PROs across all phases of the survivorship trajectory, using these insights to optimize care, improve outcomes, and ultimately enhance the quality of life for cancer survivors.

## Data Availability

No datasets were generated or analysed during the current study.

## Abbreviations

AYAs: Adolescents and young adults
BMI: Body mass index
CIPN: Chemotherapy-induced peripheral neuropathy
ECR: Eindhoven Cancer Registry
HRQoL: Health-related quality of life
NCR: Netherlands Cancer Registry
PROFILES: Patient-reported outcomes following initial treatment and long-term evaluation of survivorship
PROs: Patient-reported outcomes
QOL: Quality of life
